# The Gut Microbiota Links Dietary Polyphenols With Management of Psychiatric Mood Disorders

**DOI:** 10.3389/fnins.2019.01196

**Published:** 2019-11-05

**Authors:** Susan Westfall, Giulio Maria Pasinetti

**Affiliations:** Department of Neurology, Icahn School of Medicine at Mount Sinai, New York, NY, United States

**Keywords:** gut-brain-axis, neuroinflammation, polyphenols, resilience, probiotics, synbiotics

## Abstract

The pathophysiology of depression is multifactorial yet generally aggravated by stress and its associated physiological consequences. To effectively treat these diverse risk factors, a broad acting strategy is required and is has been suggested that gut-brain-axis signaling may play a pinnacle role in promoting resilience to several of these stress-induced changes including pathogenic load, inflammation, HPA-axis activation, oxidative stress and neurotransmitter imbalances. The gut microbiota also manages the bioaccessibility of phenolic metabolites from dietary polyphenols whose multiple beneficial properties have known therapeutic efficacy against depression. Although several potential therapeutic mechanisms of dietary polyphenols toward establishing cognitive resilience to neuropsychiatric disorders have been established, only a handful of studies have systematically identified how the interaction of the gut microbiota with dietary polyphenols can synergistically alleviate the biological signatures of depression. The current review investigates several of these potential mechanisms and how synbiotics, that combine probiotics with dietary polyphenols, may provide a novel therapeutic strategy for depression. In particular, synbiotics have the potential to alleviate neuroinflammation by modulating microglial and inflammasome activation, reduce oxidative stress and balance serotonin metabolism therefore simultaneously targeting several of the major pathological risk factors of depression. Overall, synbiotics may act as a novel therapeutic paradigm for neuropsychiatric disorders and further understanding the fundamental mechanisms of gut-brain-axis signaling will allow full utilization of the gut microbiota’s as a therapeutic tool.

## Introduction

Major depressive disorder (MDD) is a recurrent psychological disorder with numerous pathophysiological characteristics that result in prolonged periods of sadness and emptiness coupled with anhedonia, elevated anxiety and eventual cognitive dysfunction (Pellegrino et al., 2013). Depression is a significant global affliction present in more than 350 million people (World Health Organization [WHO], 2018), 16 million whom reside in the United States accounting for 6.7% of the total population (National Institute of Mental Health, 2017). Based on clinical observations, depression is defined as a multifactorial disorder with several heterogeneous neuropathological indications including reduction in the size and density of γ-amino butyric acid (GABA) neurons in the prefrontal cortex and limbic regions (Savitz et al., 2014), according to the “gliocentric theory,” abnormalities in glial density and functioning (Czeh and Nagy, 2018), imbalances in monoamine neurotransmitters in synaptic clefts (Meyer et al., 2006) and loss of hippocampal volume and neuronal loss in the hypothalamus (Manaye et al., 2005). The classical cause of depression is defined as a deficiency in noradrenaline and serotonin in the hippocampus and frontal cortex, and this remains to be a prominent hypothesis due to the efficacy of pharmacological monoaminergic reuptake blockers toward improving mood status (Taylor et al., 2005). Despite this understanding, the fundamental neurobiological changes in depression remain elusive and at present are loosely attributed to the interaction of genetic predispositions and environmental factors (Bleys et al., 2018). It is known that chronic psychological or physical stress induce a battery of depressive phenotypes through mechanisms related to abnormalities in hypothalamic-pituitary-adrenal (HPA) axis signaling including hypersecretion of C-reactive protein from the hypothalamus, impaired negative feedback of the HPA axis and hypercortisolemia (Pruessner et al., 2003). However, without a causal pathological etiology to define the manifestation of depression, therapeutic development has become rooted in the alleviation of depressive-like symptoms and such therapies remain inconsistent between patients, invoke significant side effects and result in a large proportion of patients being unresponsive to them (Kupfer, 2005).

An emerging trend in the pathology of chronic neurological diseases including depression- and anxiety-like disorders is the concept of a multifactorial causation due to multisystem abnormalities. The concept of cognitive resilience has recently gained traction as a viable therapeutic strategy for these multifactorial chronic diseases and is defined as the ability to physiologically adapt to external stresses in order to maintain normal psychological and physical functioning and avoid pathological states that can drive disease (Aburn et al., 2016). Considering that the epidemiological cause of anxiety and depression include the interaction of environmental stresses and genetic disposition in a variety of physiological systems, a treatment regime incorporating probiotics and natural polyphenols may prove to be superior compared to classical pharmacological treatments as probiotics promote the production of a diverse host of bioactive metabolites from the dietary polyphenols capable of simultaneously ameliorating multiple risk factors of depression and anxiety.

## Polyphenols in Neuropsychiatric Disorders

Dietary polyphenols expand the definition of prebiotics, which were previously believed to contain various fibrous foods that predominantly generate the short-chain fatty acids (SCFAs) propionate, acetate and butyrate following fermentation by the gut microbiota. Diets rich in SCFAs or that include prebiotics that promote SCFA production such as fructooligosaccharides (FOS), galactooligosaccharides (GOS) or inulin, have been shown to have multiple beneficial immune- and metabolic- effects that can ultimately improve cognition (Bourassa et al., 2016; Stilling et al., 2016; Dinan and Cryan, 2017). Although extremely effective at promoting neurological health, the range of SCFA activity is limited compared to the variety of polyphenols and their gut-derived metabolites making dietary polyphenols an emerging therapeutic option in neurological conditions.

Polyphenols are a broad category of heterogeneous botanicals composed of hydroxylated phenyl moieties found abundantly in fruits, tea, herbs, cereals and wine (Vinson et al., 2001). Many polyphenol-rich botanicals are considered to be adaptogenic: stress-modifying phytochemicals that increase organisms’ non-specific resistance to stress by increasing their ability to adapt and survive to external stressors and stimuli (Panossian, 2017). Due to their heterogeneous nature, individual polyphenols have distinct biological activities; however, as they are found in combination in nature, they inherently have synergistic activity that must be considered when designing polyphenolic pharmaceutical agents. Indeed, many natural occurring polyphenolic mixtures have been shown to have extensive beneficial effects on cognition and mood in both healthy and diseased subjects (Table 1).

**TABLE 1:** Effect of complex polyphenolic substances on markers of depression.

**Dietary Polyphenol**	**Contents**	**Biological Effect**	**References**
Concord Grape Juice (CGJ)	Flavanols Flavones Quercetin Phenolic Acids Proanthocyanidins Anthocyanins	In aging rats, grape juice fed *ad libitum* at concentrations of 10% enhanced cognitive performance and dopamine release while at 50%, improved motor function	Shukitt-Hale et al., 2006
		In older adults with memory decline, 6–9 ml/kg of concord grape juice for 12 weeks significantly improved cognitive function, but not depressive symptoms	Krikorian et al., 2010a
		In healthy middle-aged working women, 355 ml of CGJ consumption daily for 12 weeks significantly improved spatial memory and driving performance	Lamport et al., 2016
		In 20 healthy young adults, 230 ml of purple grape juice improved reactive time, increased calm ratings, elicited a positive effect on memory reaction time	Haskell-Ramsay et al., 2017
		A biosynthetic epicatechin metabolite derived from grapes, 3′-*O*-methyl-epicatechin-5-*O*-β-glucuronide, promotes basal synaptic transmission and long-term potentiation in hippocampal slices through mechanisms associated with CREB signaling	Wang J. et al., 2012
Cocoa	Catechins Anthocyanins Proanthocyanins Flavanols Epicatechin	Dark chocolate fed to rats exposed to air pollution of Mexico city prevented the associated neuroinflammation, COX-2 expression, IL-1β and CD14 mRNA expression in the dorsal vagal complex	Villarreal-Calderon et al., 2010
		Administration of a cocoa polyphenolic extract (22.9 mg/kg/day) to rats after heat exposure protected animals against the associated cognitive impairments as measured in the Morris Water Maze, associated with reduced free radical production by leukocytes	Rozan et al., 2007
		In healthy subjects, consumption of a dark chocolate drink mix containing 500, 250, or 0 mg of polyphenols over 30 days improved measured of mood	Pase et al., 2013
		In the Cocoa, Cognition and Aging (CoCoA) study, consumption of an enriched cocoa flavanol drink containing high (990 mg), medium (520 mg) or low (45 mg) levels of cocoa flavanols per day over 8 weeks improved cognitive function in 90 elderly adults with mild cognitive impairment in a dose-dependent manner	Desideri et al., 2012; Mastroiacovo et al., 2015
Blueberries	Anthocyanins	After 8 weeks of feeding a 2% blueberry supplements diet to aged rats, anthocyanins were found to cross the BBB and improve memory performance	Andres-Lacueva et al., 2005
		In 9 older adults consuming a wild blueberry juice for 12 weeks, improved paired associate learning and word recall was observed with a trend suggesting reduced depressive symptoms	Krikorian et al., 2010b
		In healthy older adults supplemented for 12 weeks with 30 ml of blueberry concentrate providing 387 g of anthocyanins, significant increases in brain activity were observed associated with improved working memory	Bowtell et al., 2017
		Another study demonstrated that acute administration of a flavonoid-rich blueberry extract in both young adults and children improved positive effect on mood	Khalid et al., 2017
Coffee	Flavanols Caffeic Acid Chlorogenic Acid	In aged rats, coffee at an equivalent dose of 5 cups per day, but not caffeine, improved the aged animals’ psychomotor control and working memory	Shukitt-Hale et al., 2013
		In a pilot clinical trial, decaffeinated coffee enriched with chlorogenic acids had a greater impact on cognitive performance than regular decaffeinated coffee	Cropley et al., 2012
Green Tea	Catechins (-)-epigallocatechin gallate (EGCG)	In a cross-sectional study involving 1003 elderly Japanese subjects, green tea consumption was associated with attenuated cognitive impairment	Kuriyama et al., 2006; Wang Y. et al., 2012
		In an elderly population with clinical mild cognitive impairment, 16 weeks of treatment with a combination of green tea extract with L-theanine, a protein found in green tea, improved memory and selective attention associated with elevated brain theta waves, which is an indicator of cognitive alertness	Park et al., 2011
		One study involving 27 elderly subjects showed that 2 g/day of green tea powder containing 220.2 mg of catechins did not impact cognitive impairment, despite having a positive effect on oxidative stress	Ide et al., 2016

Much of the interindividual variability of the aforementioned clinical studies can be explained by the polyphenols’ bioavailability, which is dependent on their fermentation by the gut microbiota and consequent secondary xenobiotic biotransformation in the liver. There are several comprehensive reviews exploring the prebiotic activity of polyphenols and how ingestion of polyphenols can beneficially alter the composition of the gut microbiota (Cardona et al., 2013; Westfall et al., 2018a) so this topic will not be discussed in further detail here. However, fewer studies have indicated how the specific metabolites produced by the gut microbiota from dietary polyphenol sources can impact the potential biological signature of depression.

## Microbiota and the Gut-Brain-Axis

The gut microbiota is a synergistic community of microorganisms residing in the gastrointestinal tract (GIT) composed of trillions of bacterial cells classified into thousands of species, each with a distinct metabolic profile (Qin et al., 2010). The two predominant phyla constituting approximately 98% of the gut microbiota are the Firmicutes and Bacteroidetes, with the remainder belonging to the phyla Proteobacteria, Verrucomicrobia, Fusobacteria, Cyanobacteria, Actinobacteria, and others (Backhed et al., 2004). The composition of the gut microbiota is highly amenable to diet, antibiotic usage, hygiene, pharmaceuticals and stress and changes in the composition of the gut microbiota result in dysbiosis, or imbalances in the composition of the gut microbiota and/or its metabolism (Aziz et al., 2013). Dysbiosis has been shown to influence the onset and/or progression of a battery of chronic diseases including metabolic syndrome, inflammatory bowel disease, depression, cardiovascular disease and neurodegeneration (Quigley, 2017).

The gut-brain-axis (GBA) is a bidirectional neuroendocrine system linking the GIT, including the microbiota, and the brain. The GBA consists of the enteric (ENS), peripheral (PNS) and central (CNS) nervous systems, neuroendocrine connections, humoral pathways, cytokines, neuropeptides and other signaling molecules derived from the gut microbiota itself or produced by the enterochromaffin cells in the gut epithelium in response to the gut microbiota (Mayer et al., 2014). There are several independent and distinct pathways that contribute to the GBA’s bidirectional signaling including inflammatory mediators, metabolic signaling, oxidative stress markers, stress modulators, neurohormone factors and direct neuronal communication through the vagus nerve (Kohler et al., 2016; Westfall et al., 2017).

One of the major mechanisms of GBA signaling influencing neurological health is inflammation. The gut microbiota influences inflammation in several ways beginning with management of the epithelial barrier’s integrity, constituting the host’s first line of physical defense against invading pathogens. The gut microbiota maintains the thick layer of highly glycosylated mucus on the gut epithelium that promotes the production of tissue repair factors and antimicrobial proteins (Rakoff-Nahoum et al., 2004). In addition, toll-like receptor (TLR)2 signaling, activated by various gram-positive bacterial ligands such as lipoteichoic acid found on *Lactobacillus plantarum* strains, is required for the microbiota-mediated protection of the epithelial barrier and formation of tight junctions (Podolsky et al., 2009). A healthy gut microbiota also regulates the expansion of invading pathogens, some of which harbor immune-activating ligands. Pattern recognition receptors (PRRs), specifically the TLRs and Nod-like receptors (NLRs), on host immune effector cells recognize a variety of antigens known as pathogen-associated molecular patterns (PAMPs) on bacteria, fungi, etc. that normally maintain the GIT’s basic immune tone; however, if activated in excess, initiate an innate immune response. The gut microbiota can also influence the cytokine profile of dendritic cells, which is critical to determine the fate of naïve CD4+ T helper (Th)0 cells into Th1, Th2, Th17 or regulatory T cells (Treg) in secondary lymphoid tissues (Barberi et al., 2015), which determines the inflammatory tone in the periphery and brain. It was previously shown that the ratio of Firmicutes to Bacteroidetes determines the balance of Th17 and Treg cells while *Bifidobacterium breve, B. infantis*, and *L. salivarius* were each shown to dose-dependently inhibit the differentiation and activity of early precursor dendritic cells (Round and Mazmanian, 2010). In addition, fecal transplant from inflammatory bowel disease patients into gnotobiotic mice was shown to alter the balance of gut Th17 and RAR-related orphan receptor (ROR)γT^+^ cells favoring elevated numbers of proinflammatory Th17 cells, ultimately exasperating the colitis phenotype in mice (Britton et al., 2019). Although there is little information about the specific gut bacteria that regulate the immune system, one study showed that *Bacteroides fragilis*, which produces a specific bacterial polysaccharide, directly impacts the cellular and physical maturation of the immune system including correcting T cell deficiencies and Th1/Th2 imbalances observed in germ-free mice (Mazmanian et al., 2005).

Apart from inflammatory signaling, the gut microbiota can communicate with the brain through direct nervous afferents. The ENS is the brain of the GIT governing its activity and homeostasis. From the ENS, afferent sensory pathways innervate the nucleus of the solitary tract (NTS) in the brainstem, which integrates the GIT-derived sensory information with autonomic and homeostasis-related functions in the GIT (Browning and Mendelowitz, 2003). Vagal efferents release acetylcholine to excite enteric neurons and inhibit gastric functions, which is a major contributor to symptomatic GIT dysfunctions in response to stress (Travagli et al., 2006). In addition, the vagus nerve originating in the NTS/dorsal vagal complex innervates several key visceral organs including the heart, lungs and GIT through the cholinergic system (Pavlov et al., 2003). Of particular importance, vagal efferents are known to have counter-inflammatory roles through the activation of nicotinic receptors on macrophages, downregulation of T cells and downregulation tumor necrosis factor (TNF)α production via α7-nAChR-agonistic signaling (Ghia et al., 2006). This demonstrates the complexity of the vagal connections and how dysbiosis may have a broad influence on the general inflammatory state in the body.

Finally, the gut microbiota is fundamental in managing the availability of neurotransmitters both through their synthesis in the epithelial lining and the metabolism of their precursors in the GIT lumen. An important study conducted by Asano et al. (2012) demonstrated that the gut microbiota is critical for the production of catecholamines in the luminal space. In addition, *Clostridium* ssp. are required for the biotransformation of catecholamines into their bioactive form owing to their β-glucuronidase enzymes (Asano et al., 2012). It was later determined that several microbiota species produce dopamine including *Bacillus cereus*, *B. subtilis* and *Staphylococcus aureus* (Wall et al., 2014). The gut microbiota is also critical in managing the bioavailable levels of tryptophan and consequently the synthesis of serotonin. Indeed, 95 % of all serotonin synthesis occurs in the GIT, which influences its availability in the brain (O’Mahony et al., 2015). There is also evidence suggesting that *L. plantarum* can produce acetylcholine (Stanaszek et al., 1977), *Bifidobacteria* and *Lactobacillus* spp. can produce micromolar concentrations of GABA (Barrett et al., 2012) and histamine may be produced by several gram-negative species (Devalia et al., 1989). In most of these instances, there also indications that these neurohormone producing species could invoke behavioral effects in animals. For example, the GABA-producing *L. brevis* FPA 3709 can significantly reduce depressive-like behavior in rats as effectively as the known antidepressant fluoxetine (Ko et al., 2013).

The GBA is an integrated and complex bidirectional communication system (Westfall et al., 2017) that is heavily impacted by the composition of the gut microbiota and its metabolites. Modulation of the basal GBA signaling by dietary polyphenols can have important consequences for the prevention and management of neuropsychiatric disorders by simultaneously attenuating multiple risk factors including inflammation, neuronal innervation through ENS-CNS communication and bioavailability of neurotransmitters and their precursors.

## The Gut Microbiota in Neuropsychiatric Disorders

The first indication that the gut microbiota can influence a psychiatric disorder was found with irritable bowel syndrome (IBS) (Mulak and Bonaz, 2004); however, this understanding has since been expanded to several other conditions including depression and anxiety (reviewed in Inserra et al., 2018). For example, germ-free mice have reduced anxiety compared to specific pathogen free mice, which is correlated to reduced brain-derived neurotrophic factor (BDNF) expression in the amygdala (Arentsen et al., 2015). This corroborates an earlier study that showed how antibiotic treatment could promote exploratory behavior and hippocampal expression of BDNF linking the composition of the gut microbiota directly with neurochemical and neurobehavioral effects (Bercik et al., 2011). These early animal studies have since been extended to demonstrate that the gut microbiota of depressed patients is significantly altered, potentially causatively driving symptoms of depression. In a cohort of 10 individuals with severe depression, comparative metaproteomic analyses revealed that there are significant variations in the phyla Bacteroidetes, Proteobacteria, Firmicutes, and Actinobacteria extending to altered abundances in 16 families including reduced *Bifidobacteriaceae* and *Prevotellaceae* with elevated *Enterobacteriaceae* and *Ruminococcaceae* (Chen Z. et al., 2018). A clinical study of 35 patients with major depression showed that depressive symptoms were associated with reduced gut microbiota richness and diversity while a fecal transplant from depressed patients into germ-free mice could reconstitute the depressive phenotype indicating that the gut microbiota causatively promotes depression (Kelly et al., 2016). In a similar cohort, significant variations in the phyla Firmicutes, Actinobacteria, and Bacteroidetes were observed in clinically depressed patients and again, fecal transplantation into mice could transfer the phenotype (Zheng et al., 2016). Interestingly, there are reported gender differences in the gut microbiota composition of depressed patients where female patients have a characteristic increase in the phyla Actinobacteria, male patients have reduced Bacteroidetes and both genders display disrupted Firmicutes homeostasis and elevated *Collinsella* spp. abundance (Chen J.J. et al., 2018).

There have also been intervention studies to understand how supplementation with psychobiotics, probiotics that can impact neuropsychiatric conditions, may influence depressive and anxiety phenotypes. In wild-type mice fed a western-style diet incorporating 33% fat and 49% refined carbohydrates, a phenotype resembling elevated anxiety and memory deficits was observed though prevented by treatment with *L. helveticus R0052* (Ohland et al., 2013). In a study of 124 healthy volunteers (mean age 62 years), those who consumed a mix of psychobiotics (*L. helveticus* and *B. longum*) exhibited less anxiety and depression than controls (Dinan and Cryan, 2013). In a more recent study with cohort of 79 participants with self-reported mood measures, a probiotic preparation also containing *L. helveticus* and *B. longum* did not significantly alter the mood or depression scores compared to the placebo group, however this could be from the heterogeneity, severity or chronicity of the treatment cohort (Romijn et al., 2017). In a large cohort of pregnant women, supplementation with *L. rhamnosus* HN001 lead to significantly less postpartum depression and anxiety compared to placebo controls (Slykerman et al., 2017). In another cohort of patients diagnosed with both IBS and major depression, a twice daily dose of *Bacillus coagulans* MTCC 5856 was administered and treated patients demonstrated reduced depressive phenotypes on multiple scales (Majeed et al., 2018). This accumulation of fecal transplant and psychobiotic intervention studies demonstrate that through the GBA, variations in the composition and consequently metabolism of the gut microbiota has potential therapeutic efficacy for the treatment of neuropsychiatric conditions. To amplify this effect, psychobiotics can be administered in conjunction with dietary polyphenols, as a synbiotic, increasing the production of bioactive metabolites acting on the aforementioned GBA mechanisms.

## The Gut Microbiota Increases Bioavailability of Dietary Polyphenols

### Interaction of the Gut Microbiota With Dietary Polyphenols

The interaction of gut bacteria with dietary polyphenols has a two-fold impact on health. First, dietary polyphenols act as prebiotics enhancing the growth of specific beneficial bacterial species that elicit health benefits (Cardona et al., 2013; Duenas et al., 2015; Ozdal et al., 2016). Second, autochthonous gut microbiota can increase the production of bioactive phenolic acids derived from dietary polyphenols increasing their beneficial biological activity (Espin et al., 2017). The former prebiotic effect of botanicals has been extensively described in several reviews (Cardona et al., 2013; Duenas et al., 2015; Ozdal et al., 2016) so will not be further elaborated here; however, the biotransformation of dietary polyphenols by the gut microbiota creating a diverse host of bioactive phenolic acids is a developing understanding, especially toward the promotion of cognitive resilience to depression and anxiety. The gut microbiota contains approximately 10^14^ bacterial cells, 10 times more than mammalian cells present in the human body, which contributes to its huge metabolic potential (Kardum and Glibetic, 2018). In this sense, the gut microbiota can be considered as both a metabolic and an endocrine organ that is critically important for numerous biological activities.

**FIGURE 1:**
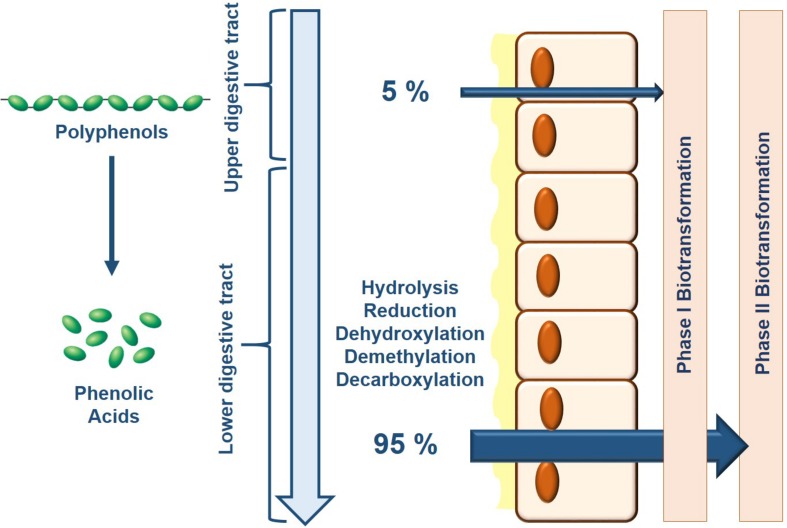
Bioavailability of dietary polyphenols is primarily determined by the composition of the gut microbiota. As consumed, only 5% of dietary polyphenols are absorbed where consequently, Phase I and Phase II biotransformation takes place in the epithelial cells and liver which generates a limited battery of bioavailable metabolites. Upon reaching the lower colon, the gut microbiota breaks down the remaining dietary polyphenols through their endogenous enzymatic activity increasing their relative bioavailability for downstream processing by Phase I and Phase II biotransformation.

Only 5–10% of dietary polyphenols are absorbed in the small intestine where they subsequently undergo phase I biotransformation (i.e., oxidation) in the endothelial cells and phase II biotransformation (i.e., conjugation) in hepatocytes liberating water-soluble conjugate metabolites (Manach et al., 2005) (Figure 1). The remaining polyphenols transit through the small intestine into the colon where the gut microbiota with their specific enzymatic makeup facilitate the bioconversion of various polyphenols and their intermediate metabolites (Braune and Blaut, 2016; Espin et al., 2017). In their natural form, dietary polyphenols are present as conjugates with sugars or organic acids that need to first be liberated before absorption. In the colon, microbial enzymes de-conjugate polyphenols producing the less-polar aglycone forms that can be either absorbed or processed though subsequent microbial reactions in the colon (Murota et al., 2018). There are three major catabolic mechanisms elicited by the gut microbiota to produce bioactive phenolic acids: hydrolysis (*O*-deglycosylations and ester hydrolysis), cleavage (C-ring cleavage, delactonization, demethylation) and reduction (dehydroxylation and double bond reduction) (reviewed in Espin et al., 2017). Indeed, several studies have identified specific enzymes in various gut microbiota that conduct these reactions; however, it must be recognized that ultimately, it is a combination of reactions conducted by several microbiota species that produce the final bioactive phenolic acids, a process known as cross-feeding (Duda-Chodak et al., 2015).

One study reported that in rats, 85% of blueberry anthocyanins reached the colon, though 69% disappear from the GIT after 4 h indicating that dietary anthocyanins are heavily metabolized by the gut microbiota (Kahle et al., 2006). Further, an anthocyanin-rich extract from black currants only demonstrated metabolic benefits in the presence of an intact gut microbiota (Esposito et al., 2015). Another study showed that mulberry anthocyanins were specifically transformed by *S. thermophilus* (46.2%) and *L. plantarum* (43.6%) into chlorogenic acid, cypto-chlorogenic acid, caffeic acid and ferulic acid: all phenolic acids with potent anti-inflammatory benefits (Cheng J.R. et al., 2016). Anthocyanins can also be broken down into protocatechuic acid, further into the bioactive phenolic acid cyanidin-3-glucoside (Vitaglione et al., 2007) and finally into 3-hydroxycinnamic acid, which has potent anti-depressive effects possibly through mechanisms implicating inflammation (Hanske et al., 2013).

Since there is high interpersonal variability in the composition of the gut microbiota, production of bioactive metabolites is also highly variable making the physiological health benefits of dietary polyphenols unpredictable in diseased populations whose display dysbiosis. As such, to standardize the physiological benefits of dietary polyphenols, they can be delivered together with probiotics (i.e., synbiotics) normalizing the production of bioactive metabolites, which can then be optimized toward having a beneficial effect. Some authors have identified that these interactions between specific gut microbial species and dietary polyphenols may have negative impacts on the host (Galati and O’Brien, 2004; Nunes et al., 2008), however the vast majority of the interactions are positive, and increase the bioavailability of the ingested polyphenols to elicit beneficial health effects.

### Synbiotics Increase the Bioavailability of Polyphenolic Metabolites Enhancing Their Biological Effect

There are only a handful of studies investigating the impact of synbiotics on cognition, and even fewer that utilize a polyphenolic prebiotic. In general, clinical studies with synbiotics are inconclusive as there are a broad cohort diversities and inadequate regulation of treatment regimens as was demonstrated with meta-analyses on IBS patients (Ford et al., 2018), ulcerative colitis (Asto et al., 2019), and diabetes (Zheng et al., 2019). Nevertheless, a few notable studies have been conducted to date demonstrating the potential of synbiotics to act as powerful therapeutic agents in the management of neurological disorders. In a study of a healthy elderly population, patients were separated into either placebo or synbiotic groups where the latter were exposed to two daily doses of a FOS and a probiotic formulation containing *L. paracasei*, *L. rhamnosus, L. acidophilus*, and *B. lactis.* Although there were no significant differences in the depression scores, the synbiotic did improve inflammatory markers of the healthy elderly individuals, notably with an increase in the anti-inflammatory IL-10 cytokine, associated to improvements in cognition (Louzada and Ribeiro, 2018). In a separate study, a cohort of 75 hemodialysis patients were administered a synbiotic containing *L. acidophilus, B. bifidum, B. lactis*, and *B. longum* with the prebiotics FOS, GOS and inulin. In a subset of patients with depressive symptoms, the synbiotic significantly reduced the depressive score compared to both the probiotic-alone and placebo groups, which correlated to an increase in BDNF serum levels (Haghighat et al., 2019). In one comprehensive animal study, the effects of probiotic (*L. paracasei*), prebiotic (xylooligosaccharide) and synbiotic treatment on chronic high-fat diet (HFD) induced obesity and insulin resistance was evaluated including measures of the associated HFD-induced cognitive decline. Interestingly, all treatment groups reduced HFD-associated inflammation, hippocampal oxidative stress, apoptosis and microglial activation. Although there were no statistical differences between the prebiotic or probiotic groups with the synbiotic, the synbiotic did have a trending beneficial effect on multiple measures of hippocampal activity including dendritic spine density, soma area and apoptosis measures, which could be potentially amplified with the use of a more complex prebiotic formula such as a polyphenol (Chunchai et al., 2018). Although interesting, these synbiotic studies only utilize the traditional fiber-based prebiotics, and to the author’s knowledge, only one study to date has tested how a polyphenol-rich synbiotic can affect multiple markers of cognition, using an Alzheimer’s Disease (AD) model in *Drosophila*. In this study, a synbiotic that was previously shown to enhance production of polyphenolic metabolites (Westfall et al., 2018a) and promote longevity in *Drosophila melanogaster* (Westfall et al., 2018b) was shown to rescue the AD phenotype in humanized transgenic *Drosophila* (Westfall et al., 2019). In particular, the synbiotic-derived metabolites provided potent anti-inflammatory and antioxidant activity while reestablishing metabolic homeostasis. Of particular interest, when considering all of the AD risk factors as a whole, the synbiotic consistently rescued all of the risk factors to a greater or same extent as its components establishing its combinatorial activity. Despite the lack of studies truly investigating the combinatorial action of synbiotics containing polyphenolic prebiotics, below is a description of potential polyphenolic precursors that are known to require the gut microbiota to produce its full extent of metabolites and elicit potential beneficial effects on cognition and mechanisms associated with depression.

Roasted green coffee beans contain a high level of hydroxycinnamates, which are partially bioavailable yet extensively metabolized, mainly by the colonic microbiota. In subjects who drank a roasted coffee blend containing 269.5 mg of chlorogenic acids, the majority of metabolites in the urine (75.7%), composed of dihydrohydroxycinnamic acids and feruloylglycine, were of colonic origin (Gomez-Juaristi et al., 2018b). The same group identified that the polyphenolic-rich yerba mate was mainly metabolized by the colonic microbiota with up to 81 % of the metabolites composed of dihydroferulic acid, dihydrocaffeic acid and dihydrocoumaric acids (Gomez-Juaristi et al., 2018a). Another example is ester hydrolysis of chlorogenic acid to release caffeic acid, which was determined to be carried out by *B. animalis* by a specific enzyme identified as Balat_0669 (Raimondi et al., 2015).

The urolithins are an important class of bioactive microbial metabolites derived from ellagitannin and ellagic acid precursors. Several bacterial species have been identified that produce their intermediate metabolites including *Gordonibacter urolithinfaciens* and *G. pamelaeae* (Selma et al., 2014); however it has recently been shown that a new class of microbiota species, the *Eggerthellaceae* family, is essential to produce the final urolithin metabolite isourolithin A (Selma et al., 2017; Beltran et al., 2018). It is well known that the microbial-derived urolithins have potent anti-inflammatory effects. In human colonic fibroblasts, urolithins, but not their ellagitannin precursor, inhibited nuclear factor kappa-light-chain-enhancer of activated B cells (NF-κB) translocation into the nucleus and consequent activation of downstream inflammatory events (Gonzalez-Sarrias et al., 2010). These effects were extended to indicate the microbial-derived urolithins are neuroprotective and pomegranate’s anti- AD’s neuroprotective ability was attributed to the production of microbial-derived urolithins (Yuan et al., 2016).

Catechins and epicatechins are major constituents of grape seed extracts and the production of their bioactive metabolites is dependent on the presence of the microbiota (Ou et al., 2014). In two grape-seed extracts containing either 70% monomers and 28% procyanidins or 21% monomers and 78% procyanidins, the growth of *Lactobacillus* and *Enterococcus* spp. were elevated while the *Clostridium histolyticum* group was inhibited indicating that specific gut bacteria are responsible for the metabolism of grape seed flavan-3-ols. These changes in the gut microbiota were associated with increases in 4-hydroxyphenylacetic acid, phenylpropionic acid, phenylacetic acid and 4-hydroxybenzoic acid (Cueva et al., 2013). *Eggerthella lenta* JCM 9979 was shown to facilitate the C-ring cleavage of epicatechins and catechins and the subsequent 4’-dehydroxylation to produce different intermediate metabolites (Takagaki and Nanjo, 2015). *L. plantarum* IFPL935 was found to be important in the first step of catechin and procyanidin catabolism involving ring fission, however did not impact the production of phenolic metabolites unless in the context of a complete microbiota indicating that there is another microbe using the metabolic intermediate of *L. plantarum* to produce the bioactive metabolites (Barroso et al., 2013).

The natural flavonoid, quercetin, is also heavily processed by the gut microbiota. In an *in vitro* anaerobic fermentation model, the fecal microbiota was shown to deconjugate rutin, isoquercetin and a mixture of quercetin glucuronides through deglycosylation, ring fission and dehydroxylation reactions to produce the metabolites 3,4-dihydrophenylacetic acid and 3-hydroxyphenylacetic acid (Aura et al., 2002). In an elderly Japanese population, interindividual variations in quercetin concentrations with respect to fecal microbiota compositions were observed and the level of quercetin consumption was negatively correlated with the abundance of *Sutterellaceae* and *Oscillospiraceae* spp. and positively correlated with the families *Fusobacteriaceae* and *Enterobacteriaceae* (Tamura et al., 2017).

When considering neurological diseases, it is important not only to understand the bioavailability of the phenolic acids in the colon and plasma, but also in the brain where their activity is warranted. Grape seed polyphenol extract (GSPE) is a rich source of flavan-3-ols including catechin, epicatechin, and anthocyanins which were shown to produce a variety of bioavailable phenolic acids both in the plasma and brain (Ho et al., 2013; Wang et al., 2015). Importantly, the production of bioactive phenolic acids derived from the anthocyanin-rich GSPE is dependent on the microbiota, and two of the microbiota-derived metabolites 3-hydroxybenzoic acid and 3-(3′-hydroxyphenyl)propionic acid accumulate in micromolar concentrations in the brain where they can interfere with the assembly of amyloid-beta peptides (Wang et al., 2015). We also found that after moderate wine consumption in rats, there is an accumulation of the polyphenol metabolite quercetin-3-*O*-glucuronide in the brain, which specifically reduced the generation of amyloid-beta from primary neuron cultures generated from the Tg2576 AD mouse model (Ho et al., 2013). At the same time, this metabolite significantly improved AD-type deficits in hippocampal formation basal synaptic transmission. Going further, a Bioactive Dietary Polyphenol Preparation (BDPP) containing GSPE, concord grape juice and resveratrol was shown to attenuate sleep deprivation-induced contextual memory deficits. Supplementation of BDPP lead to the accumulation of malvidin-3-*O*-glucoside and quercetin-3-*O*-glucuronide in the brain where the former activated target of rapamycin (mTOR) signaling and the latter cAMP response element-binding protein (CREB) signaling (Zhao et al., 2015). Combining malvidin-3′-*O*-glucoside with another BDPP metabolite, dihydrocaffeic acid (DHCA) significantly promoted cognitive resilience to stress-induced depression by modulating neuronal plasticity and peripheral inflammation in stressed rats. Mechanistically, DHCA was shown to inhibit DNA methylation on CpG-rich interleukin (IL)-6 sequences while malvidin-glucoside increased histone acetylation of the regulatory sequences that modulate synaptic plasticity (Wang J. et al., 2018).

Despite the small number of studies on the specific activity of synbiotics including a polyphenol-rich prebiotic on the specific mechanisms of depression, it can be concluded that the gut microbiota is essential for producing the full battery of plasma- and brain-bioactive polyphenolic metabolites that have potential neuroprotective activity. Below is a description of how some of the individual microbial-derived polyphenolic metabolites can impact different mechanisms that lead depression, strengthening the argument for the use of synbiotics as depressive therapies.

## Gut-Brain-Axis Mechanisms Linking Polyphenolic Microbial Metabolism to Depression

### Gut Microbiota-Anti-inflammatory Effects Modulate Depression

#### Neuroinflammation Implicates Neurological Changes in MDD

Chronic neuroinflammation is a major risk factor for neurological diseases as it leads to changes in brain structure and synaptic plasticity resulting in neural deficits (Maes, 1999). Neuroinflammation also modulates neurotransmitter concentrations by upregulating monoamine transporters, reducing synaptic reuptake of monoamines and reducing monoamine synthesis by decreasing tetrahydrobiopterin availability, a cofactor necessary for both tyrosine and tryptophan hydroxylases (Miller and Raison, 2016). The elevated release of cytokines observed with neuroinflammation also drives glutamatergic neurotransmission inducing excitotoxicity and consequently neuronal death (Haroon et al., 2014). Although neuroinflammation is not specific to depression, it does account for a large part of its pathophysiology and anti-inflammatory medication has been successful in alleviating some depressive symptoms (Kohler et al., 2014; Ebada, 2017). As previously described, stress is a major risk factor for depression and neuroinflammation could explain, in part, how stress induces the psychological impairment characteristic of depression. Neuroinflammation, especially elevation in interferon(IFN)-α and IL-6 cytokines, inhibits negative-feedback regulation of the HPA axis, therefore maintaining hypercortisolemia in the context of chronic stress. This elevated glucocorticoid release exasperates the stress response and reduces sensitivity of peripheral immune cells to anti-inflammatory feedback (Frank et al., 2012). Hence, neuroinflammation, when coupled with a reduction in neuroprotection and neuronal repair due to elevated glucocorticoid levels, may be among the initial pathological markers of depression and controlling neuroinflammation with a dietary regime incorporating synbiotics could be a viable prophylactic approach for preventing the onset and/or progression of depression.

Several associations between inflammatory conditions and susceptibility to depression have been made. Patients with rheumatoid arthritis, cancer, autoimmune diseases or other chronic inflammatory conditions are predisposed to depression (Pollak and Yirmiya, 2002). Also, several inflammatory markers have also been used as diagnostic indicators of MDD (Musselman et al., 2001; Vogelzangs et al., 2012). Based on post-mortem studies, depressed patients were found to have area-dependent elevation in proinflammatory cytokine mRNA and protein expression, which is linked to the prominent downregulation in both number and density of oligodendrocytes in areas associated with the depressive phenotype (Mechawar and Savitz, 2016). Depressed suicide completers in particular have elevated mRNA and protein levels of TNF-α, IL-1β and IL-6 in the prefrontal cortex (Pandey et al., 2012), consistent with elevated TLR expression in macrophages and microglia in the corresponding areas (Pandey, 2017).

In depressed patients, there is also evidence of increased blood-brain-barrier (BBB) permeability (Bechter et al., 2010) and through a compromised BBB, cytokines and chemokines infiltrate the CNS, stimulating microglia and astrocyte activation (Wohleb et al., 2016). In a mouse preclinical repeated social stress model, BBB impairment in the nucleus accumbens region was observed in stress-susceptible mice and confirmed in postmortem depressed patients as observed with decreased expression of a key tight-junction protein, claudin 5. This loss of barrier integrity was associated with elevated infiltration of cytokines and subsequent expression of depression-like behaviors (Menard et al., 2017). The circumventricular organs (CVOs) border the brain’s ventricular spaces and lack the typical tight junction integrity of the BBB (Petrov et al., 1994). TLRs on macrophage-like cells in the CVOs are uniquely activated by systemic inflammation and can increase production of proinflammatory mediators. One CVO in particular, the area postrema, is highly interconnected with the nucleus of the solitary tract and dorsal motor nucleus of the vagus nerve (Maolood and Meister, 2009), directly linking inflammatory signals from the GIT with compromised BBB integrity through the CVOs. A compromised BBB also allows the infiltration of peripheral immune cells, including monocytes, dendritic cells and T lymphocytes, into the brain (Najjar et al., 2013). Stress can bias myeloid lineage cells to increase their trafficking ability promoting their increased infiltration into the brain parenchyma (Wohleb et al., 2013). This elevated trafficking could be due to elevated expression of key immune adhesion molecules in specific brain regions after stress. As such both intercellular adhesion molecule (ICAM)-1 and vascular cell adhesion molecule (VCAM)-1 were observed to be increased on endothelial cells in the prefrontal cortex and hypothalamus following a repeat social defeat paradigm in mice, which parallels the patterns of macrophage trafficking and microglial activation in the brain. Once in the brain, these infiltrated monocytes alter behavior and promote microglial reactivity exasperating the neuroinflammatory response (Ataka et al., 2013).

#### Compromised Gut-Brain-Axis Signaling Instigates Neuroinflammation

A stable and healthy commensal microbiota plays a cardinal role in maintaining the host’s immune status (Bercik et al., 2011). On one side, the gut microbiota is immunomodulatory (Round and Mazmanian, 2009; El Aidy et al., 2015), while on the other side, the immune system works to shape the composition and diversity of the intestinal microbiota (Hooper et al., 2012). Incorporation of dietary polyphenols dually impacts the gut microbiota-mediated immunomodulatory effects as they alter both the composition of the gut microbiota with their prebiotic activity while providing precursors for the production of many microbial-derived metabolites that have protective influences on the gut barrier, the first line of immune modulation.

As a first line of defense, the gut microbiota maintains the integrity of the intestinal epithelium, which prevents infiltration of bacteria and other immune-triggering substances into the host’s circulation. The microbiota accomplishes this through a variety of mechanisms including maintaining tight junction proteins, production of the mucus layer and secretion of antibactericial proteins and factors, such as IgA, to fend off invading pathogens (Daulatzai, 2014). A major gut-derived mediator of inflammation is lipopolysaccharide (LPS), which through TLR4 signaling activates NF-κB-mediated transcription of proinflammatory cytokines from monocytes and macrophages (Tanti et al., 2012). GIT-mediated inflammation stimulates barrier breakdown and consequently, elevated infiltration of LPS and other bacterial components. One study specifically showed that elevated GIT permeability with an increased translocation of LPS from gram-negative bacteria plays a significant role in the pathophysiology of MDD (Maes et al., 2008). LPS dose-dependently increases IL-1β levels in the dorsal vagal complex, as well as in the hypothalamus, hippocampus, cerebellum, neocortex and pituitary gland (Hansen et al., 2000). Indeed, a single injection of LPS is associated with elevated systemic and central inflammatory mediators and significant cognitive deficits (Kahn et al., 2012), while chronic LPS injections over 5 days in female mice at 1 month intervals induces chronic anhedonia (Kubera et al., 2013).

Interestingly, the gut microbiota has also been associated with deficits in BBB integrity. Germ-free mice have increased BBB permeability and lower expression of occludin and claudin-5 in different brain regions implicated in depression including the frontal cortex, hippocampus and the striatum (Braniste et al., 2014). This group also confirmed that mono-colonization of germ-free mice with the butyrate producing *Clostridium tyrobutyricum* elevated occludin expression in the frontal cortex and hippocampus while reducing BBB permeability (Braniste et al., 2014).

The vagus nerve is also an important player in the bidirectional communication between the gut microbiota and the brain as it monitors the physiological homeostasis of the GIT and connects it to the cognitive and emotional centers in the CNS (Carabotti et al., 2015). One of the major functions of vagal afferents is activation of the HPA axis, which coordinates the adaptive responses of the organism with external stressors, and directly links the health of the microbiota and GIT to depressive phenotypes (Howland, 2014). The vagus nerve also implements an inflammatory reflex where pathogenic species that induce proinflammatory cytokines can activate afferent sensory vagal fibers synapsing in the nucleus tractus solitarius. Efferent vagal signals communicate with the periphery and HPA axis to reduce inflammatory tone by inhibiting the release of TNF-α by splenic nerves (Breit et al., 2018). Notably, there are also receptors on the vagal afferents for various cytokines and TLRs, which can initiate the synthesis and release of inflammatory cytokines from cells within the CNS (Dantzer et al., 2008).

#### Gut-Derived Polyphenols Reduce Neuroinflammation

There is ample information supporting the anti-inflammatory activity of polyphenols and their metabolites in the context of neurological disorders. However, most studies neglect to integrate the importance of the gut microbiota in producing the polyphenolic bioactives thereby underestimating their full anti-inflammatory potential. Ferulic acid is a hydroxycinnaminic acid produced as a microbial metabolite from several *Lactobacillus* species (Tomaro-Duchesneau et al., 2012) and the production of many of its bioactive metabolic products (dihydroferulic acid or vanillic acid) is dependent on the gut microbiota. Ferulic acid has been shown to have potent implications in depression. *Ligusticum officinale* is an anti-inflammatory plant used in oriental medicine which is rich in ferulic acid and potently can attenuate NF-κB activation in BV2 microglial cells following LPS stimulation (Zeni et al., 2017). In another study utilizing a defeat stress paradigm, ferulic acid at 1 mg/kg reduced oxidative stress and neuroinflammatory markers in the blood, hippocampus and cerebral cortex of mice (Lenzi et al., 2015). In an outbred ICR mouse model, ferulic acid, in combination with the bioavailability enhancer piperine, reduced immobility in the tail suspension and forced swim test by 60% possibly by inhibiting monoamine oxidase activity in the frontal cortex, hippocampus and hypothalamus (Li et al., 2015). Going further, in a model of chronic unpredictable mild stress, ferulic acid ameliorated depressive-like behaviors possibly through the upregulation of BDNF, postsynaptic protein PSD95 levels, and synapsin I in the prefrontal cortex and hippocampus (Liu Y.M. et al., 2017).

Epicatechin, catechin and the proanthocyanidins are the main flavan-3-ols metabolized by the gut microbiota that also elicit beneficial anti-inflammatory effects. Catechin pretreatment to the chemotherapeutic agent Doxorubicin in rats dose-dependently prevented neurodegeneration while at 100 mg/kg, reduced memory deficits by decreasing oxidative stress, acetylcholinesterase activity and neuroinflammation in the hippocampus (Cheruku et al., 2018). In a rat model of traumatic brain injury, catechin treatment was shown to be neuroprotective by dually protecting both BBB integrity and excessive neuroinflammation (Jiang et al., 2017). A major green tea catechin, (-)-epigallocatechin gallate (EGCG) has many neuroprotective abilities, neuroinflammation being just one of them. Pretreatment of outbred ICR mice with EGCG for 3 weeks (1.5 and 3 mg/kg/day) prior to LPS injection for 7 days prevented the LPS-induced memory impairment and apoptotic neuronal cell death. This included preventing astrogliosis associated with the LPS injections and the consequent production of inflammatory mediators (Lee et al., 2013). In a similar model, EGCG rescued LPS-induced inhibition of adult neurogenesis by restoring proliferation and differentiation of neural stem cells in the dentate gyrus and modulating neuroinflammation through the TLR4-NFκB pathway (Seong et al., 2016).

There is also ample evidence that quercetin can reduce neuroinflammation and as previously indicated, the urolithin metabolic products derived from quercetin have more bioactivity than quercetin itself. In a rat cardiopulmonary resuscitation model of depression, quercetin inhibited ROS generation, neuroinflammation and metalloproteinase-2 protein expression corresponding to recovering left ventricular ejection fraction reduced by the depression paradigm (Wang D. et al., 2018). Mice undergoing chronic unpredictable stress for 21 days were simultaneously treated with 30 mg/kg of quercetin, which alleviated both anxiety and depression behavioral dysfunctions. Simultaneously, quercetin treatment reduced the stress-induced elevation in oxidative stress markers and proinflammatory markers (Mehta et al., 2017). Adriamycin is a chemotherapeutic agent that induces depression- and anxiety-like behaviors in rats. Quercetin (60 mg/kg), alleviated the anxiety and depressive behaviors while attenuating brain oxidative stress and suppressing the excessive corticosterone induction in rats treated with Adriamycin (Merzoug et al., 2014). Quercetin was also shown to reduce depressive behavioral deficits in olfactory bulbectomized rats by simultaneously reducing the oxidative, inflammatory and stress-induced changes in the cerebral cortex and hippocampus. This group suggested that quercetin may elicit its neuroprotective effects through a microglial inhibitory pathway as subclinical amounts of quercetin potentiated the activity of minocycline, a known microglial inhibitor (Rinwa and Kumar, 2013).

The known anti-inflammatory action of resveratrol has been translated to be beneficial in various neuroinflammatory models of depression. Resveratrol inhibits several proinflammatory mediators, modifies eicosanoid synthesis and inhibits enzymes including cyclooxygenase (COX)2, NF-κB, AP-1, TNFα, IL-6 and vascular endothelial growth factor (VEGF) (Namasivayam, 2011). In a social defeat paradigm, resveratrol at 30 mg/kg body weight per day blocked neuroinflammation in the locus coeruleus, but not neurotransmitter release, associated with reduced anhedonia to the sucrose preference test (Finnell et al., 2017).

Caffeic acid is among the main constituents in coffee that have been shown *in vitro* and *in vivo* to have beneficial effects in modulating neuroinflammation (Hall et al., 2015). The derivative of caffeic acid, caffeic acid phenethyl ester, also has a battery of neuroprotective activities including anti-inflammatory and immunomodulatory properties (Noelker et al., 2005) whose production is dependent on the gut microbiota (Peppercorn and Goldman, 1971). Indeed, a negative correlation between the level of coffee consumption and depression has been recorded (Pham et al., 2014). In mice, caffeic acid (4 mg/kg) was shown to have antidepressant-like activity independent of monoamine transduction suggesting that caffeic acid works through a non-monoameringeric system (Takeda et al., 2002). Caffeic acid, at a dose of 30 mg/kg body weight in mice, prophylactically inhibited LPS-induced sickness behavior specifically by reducing cytokine production in serum and brain thus eliciting a protective effect against neuropathologies associated with depression (Basu Mallik et al., 2016).

The anti-inflammatory activity of polyphenols is well known, albeit interesting as a potential therapeutic application in depression. The activity of polyphenols, however, may only be considered in the context of the gut microbiota. As demonstrated previously, the diversity and richness of the gut microbiota is critical to determine the bioavailability of dietary polyphenols, which in their parent form, remain relatively inactive. Likewise, combining dietary polyphenols with optimized probiotic formulation as a synbiotic, would increase the production of specific metabolites whose anti-inflammatory mechanisms could be elucidate and optimized for the treatment of depression.

### Activated Microglia Impact Depressive Neuropathology

Microglia are the principle immune mediators in the CNS and their improper activation is associated with neuroinflammation and clinical psychiatric phenotypes (Steiner et al., 2008). In a resting state, microglia conduct immunosurveillance, mediating brain homeostasis and innate immune responses against a range of pathogenic insults primarily through phagocytosis (Hanisch and Kettenmann, 2007). However, under conditions of stress or elevated peripheral inflammation, the microglia transform into an activated state where there is an upregulation of the major histocompatibility complexes (MHCs) and complement receptors stimulating the production of large amounts of inflammatory cytokines and chemokines (Hanisch and Kettenmann, 2007).

Recently, depression has been described as a microglia-associated disorder with many depressed patients suffering from excessive microglial activation (Yirmiya et al., 2015). In preclinical depression models following a stress paradigm, elevated numbers of activated microglia in the hippocampus, prefrontal cortex, nucleus accumbens and amygdala have been reported (Biesmans et al., 2013; Hinwood et al., 2013). In rats subjected to a mild stress paradigm for 12 weeks as a model of depression, significant microglial activation in the hippocampus was observed (Wang J. et al., 2018). In suicide completers, there is evidence of elevated microgliosis (Steiner et al., 2008, 2011), which has been confirmed with postmortem enrichment of the microglial ionized calcium binding adaptor molecular (IBA)1 marker in the dorsal anterior cingulate cortex of depressed suicide victims (Torres-Platas et al., 2014). In addition, an association between reactive microglia and major depression was shown in a positron emission tomography study demonstrating that the density of translocator protein-18, a mitochondrial protein expressed almost exclusively in activated microglia, was significantly elevated by 30% in the prefrontal cortex, anterior cingulate cortex and insula of patients with major depression (Setiawan et al., 2015). This was confirmed in a recent study in mild to major depression patients where translocator protein 18 was more highly expressed in the anterior cingulate cortex and insula of major depression patients with suicidal thoughts (Holmes et al., 2018).

#### Gut-Brain-Axis Signaling Abrogates Microglial Activation

The first study showing that the gut microbiota can influence microglial dynamics was conducted by Erny et al. (2015), which showed distinct variations in the microglial transcriptomes of germ-free versus specific pathogen free mice. In particular, many genes involved in cellular activation were down-regulated in the microglia of germ-free animals while flow cytometry analyses indicated that these microglia were immature. Further, this group showed that chronic treatment with SCFAs could reverse the microglial immaturity and malformation observed in germ-free mice indicating the importance of microbial-derived metabolites in shaping the microglial responses (Erny et al., 2015). One study showed that repeated treatment of sodium butyrate attenuated LPS-induced depressive behaviors while simultaneously attenuating microglial activation in the hippocampus, possibly through epigenetic regulation of various promoter elements (Yamawaki et al., 2018). Another study also compared microglial activation between conventional and germ-free mice subjected to a LPS stressor. Using a cytometric bead array analysis from hippocampal and prefrontal cortex samples, germ-free mice demonstrated attenuated production of cytokines in both these areas, which correlated to the observed increase in microglial activation in conventional, but not germ-free mice. Further, the microglia in germ-free mice lacked MHCII markers, CD44 and CD62L, confirming their inability to be stimulated (Campos et al., 2016).

#### Microbial-Derived Polyphenolic Metabolites Inhibit Microglial Activation

Phenolic acids produced by the gut microbiota also modulate microglial activation. In the AD APP/PS1 mouse model, a pomegranate extract was shown to reduce microgliosis and amyloid-beta plaque deposition in association with reduced anxiety-like behavior and increased memory performance. This effect was attributed to two polyphenolic compounds, punicalagin and ellagic acid, and likely its bioactive microbial-derived metabolite EGCG (Rojanathammanee et al., 2013). Similar to EGCG, resveratrol was shown in neuron-glial primary cultures to inhibit LPS-induced microglial activation and subsequent production of TNFα, nitric oxide and IL-1β likely through modulation of inflammasome signaling (Zhang et al., 2013). In a follow up study, resveratrol reduced hypoxia-induced microglial activation in BV-2 cells, consequently reducing proinflammatory factor release by inhibiting hypoxia-induced NF-κB inhibitor (IkB)-α degradation (Zhang et al., 2015). Chronic constriction injury causes significant glial activation and neuroinflammation in the spinal trigeminal nucleus. Resveratrol treatment after the constriction injury showed an inhibitory effect on the associated microglia and astrocyte activation while reducing the production of inflammatory cytokines through a mechanism implicating MAPK activation (Yang et al., 2016). Quercetin invokes a dose-dependent decrease in nitric oxide production in BV2 microglial cells 1 h prior to LPS treatment. Mechanistically, the authors observed that quercetin suppressed cPLA2 phosphorylation, an activity that was shown to prevent microglia-induced neurotoxicity in differentiated SH-SY5Y neuroblast cells (Chuang et al., 2016). Quercetin was also shown to inhibit obesity-induced hypothalamic inflammation by inhibiting microglia-mediated inflammatory responses, likely through mechanisms involving heme oxygenase induction. These results were verified *in vivo* where microglial activation markers in the hypothalamus of high fat diet fed obese mice were reduced in quercetin-supplemented animals (Yang et al., 2017). Various anthocyanin-rich extracts, particularly from the purple basal, were also shown to attenuate nitrite release from microglial cells stimulated by LPS (Strathearn et al., 2014). Anthocyanins inhibit LPS-induced microglial activation in BV2 microglial cells by inhibiting NF-κB translocation into the nucleus and consequently cytokine release including nitric oxide and prostaglandin E2 release (Jeong et al., 2013).

Similar to the investigation on the anti-inflammatory activity of polyphenolic metabolites, many of the dietary polyphenols discussed for the management of microglial activation require the activity of the gut microbiota to produce the appropriate bioactive metabolites. With an appropriately designed synbiotic formulation, multiple bioactive polyphenolic metabolites may be produced with multiple actions promoting neuroprotection against exasperated microglial activation, consequently protecting against depressive-like phenotypes.

### Inflammasome Activity Drives Neuroinflammation in Depression

The importance of the inflammasome and sterile inflammation in translating psychological stressful stimuli into neuroinflammatory responses has become recently recognized (Herman and Pasinetti, 2018). Pharmacological inhibition (Zhang et al., 2015) or genetic depletion (Iwata et al., 2016) of the inflammasome’s assembly abolishes the depressive phenotype in response to various stress models. There are several inflammasome complexes in the body; however, the nod-like receptor pyrin containing 3 inflammasome (NLRP3), implicated specifically in caspase-1 activation, is found predominantly in the microglia under conditions of mild chronic stress (Pan et al., 2014), but can also be induced in neurons under conditions of severe stress (Zendedel et al., 2016). Indeed, NLRP3 gene expression was found elevated in PBMCs of patients with major depression corresponding with elevated serum levels of IL-1β and IL-18, supporting the clinical applicability of inflammasome activation in depression.

The inflammasome can be activated through sterile inflammation making the inflammasome an intracellular sensor to cellular stress and damage instead of direct pathogenic load. Canonical inflammasome activation requires two activating signals. The first signal stimulates the transcription of *Nlrp3*, *IL-1β* and *IL-18* proinflammatory cytokines and is under the control of the PRRs, TLR or NLR, and the subsequent activation of the NF-κB transcriptional program. As such, damaged neurons or psychological stressors release danger associated molecular patterns (DAMPs) including high mobility group box 1 (HMGB1), mtDNA, ATP and the S100 proteins which trigger TLR-associated pathways and present a maj or risk factor for depression (Fleshner et al., 2017). Each DAMP has a different affinity for either TLRs, or other PRRs such as RAGE (receptor of advanced glycation end products) or P2X7 (reviewed in Franklin et al., 2018) that leads to the same downstream inflammatory cascades including assembly and activity of the inflammasome. The second signal, such as ATP release, instigates assembly of the NLRP3 multimeric complex including recruitment of the apoptosis speck-like (ASC) protein and pro-caspase-1 (Lechtenberg et al., 2014). The assembled proteasome is responsible for the catalytic cleavage of pro-IL-1β and pro-IL-18 by activated caspase-1 leading to inflammatory-driven cellular damage, autophagy and pyroptosis (Gurung et al., 2014).

The stress-induced production of IL-1β is critical for the development of depressive-like behaviors. In a chronic unpredictable stress model in rats, IL-1β mRNA and protein levels produced from inflammasome activation were found elevated in the prefrontal cortex, but not in the serum or CSF (Pan et al., 2014). Interestingly, *Nlrp3*-null mice are resilient to restraint stress-induced depressive-like behaviors including the associated microglial activation or reduced hippocampal neurogenesis (Alcocer-Gomez et al., 2016). Following a foot-shock paradigm, HMGB1 was found to be specifically upregulated in the hippocampus and associated with elevated chemokine and cytokine production (Cheng Y. et al., 2016). Similarly, S100b is elevated in the plasma of major depression patients, and overexpression of S100 is associated with depressive-like behaviors observed with the forced swim test in mice (Stroth and Svenningsson, 2015).

#### The Gut Microbiota and Related Polyphenolic Metabolites Regulate Inflammasome Activation

**FIGURE 2:**
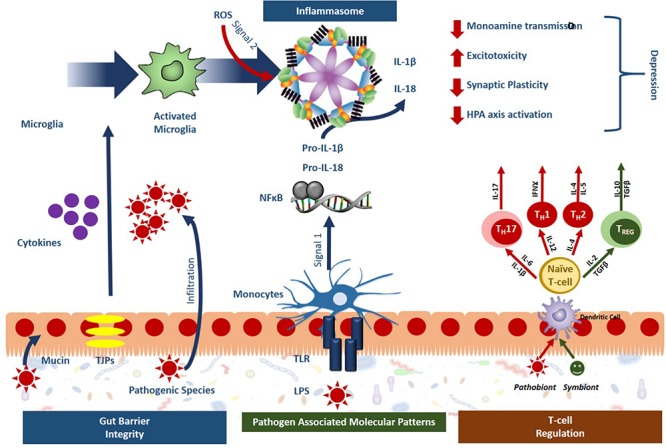
Gut microbiota activated inflammasome signaling leads to phenotypes specific to depression. Sterile inflammatory responses involving inflammasome signaling is a key mechanism of stress-induced depression and the gut microbiota has been shown to impact inflammasome signaling at several levels. Symbionts, or beneficial bacteria, have been shown to promote gut barrier integrity, and guide T-cell regulation toward an anti-inflammatory phenotype (green). Pathobionts, or bacteria with a negative effect, have the opposite effect, compromising the gut barrier integrity, activating immune response through the release of pathogen associated molecular patterns (PAMPs) and pushing T cell development toward a proinflammatory state (red). Overall, these effects lead to inflammasome activation in the periphery and the microglia that ultimately promote the development of depression.

Recently, the microbiota-inflammasome hypothesis of major depression was proposed (Figure 2) (Inserra et al., 2018). This theory suggests that there is a feedback loop where the gut microbiota-induced production of peripheral inflammation reduces the integrity of the BBB leading to inflammasome activation and consequently, imparts depressive symptoms while simultaneously disrupting the composition of the gut microbiota. A variety of microbial pathogens that can activate the NLRP3 inflammasome have been identified including *Salmonella typhimurium*, *Escherichia coli*, and *Shigella flexneri* (Brodsky and Monack, 2009); yet the mechanisms that drive this activation remain to be fully characterized. The gut bacteria can either directly activate the inflammasome, or indirectly. Through direct activation, inflammasome receptors will recognize bacterial antigens instigating the canonical inflammatory cascades while indirect activation involves sensing changes in the host’s response to infection known as “patterns of pathogenicity.” The latter includes changes in oxidative stress, potassium efflux or lysosomal destabilization (Storek and Monack, 2015) which can be sensed by the sterile inflammatory response. In one study, specific gut microbiota species were shown to stimulate IL-1β release through inflammasome signaling following spinal cord injury including the *Enterobacteriaceae* family and in particular, the pathobiont *Proteus mirabilis*. This study suggested that these selective members of the gut microbiota could stimulate newly recruited monocytes to induce NLRP3-dependent IL-1β release, promoting inflammation in the intestine and further studies may demonstrate their importance in the depressive clinical phenotypes (Seo et al., 2015).

There are several indications that polyphenol supplementation can reduce inflammasome activation. In line with its aforementioned anti-inflammatory activities, EGCG has been shown to impact inflammasome signaling in multiple models. In a contrast-induced model of renal failure, EGCG downregulated *Nlrp3* gene expression through a pathway involving a known inflammatory regulator heme oxygenase-1 (Gao et al., 2016). In another study, prophylactic EGCG treatment attenuated lupus nephritis symptoms and several inflammatory pathological targets leading to tangible preclinical benefits (Tsai et al., 2011). In endothelial cells, palmitate-induced oxidative stress lead to the strong upregulation of the NLRP3 inflammasome associated IL-1β release and apoptosis, an effect that was ameliorated by EGCG supplementation through mechanisms involving AMPK signaling (Wu et al., 2014).

Quercetin also shows promising ability to attenuate inflammasome-induced inflammation. In a spinal cord injury model in rats, quercetin significantly decreased ROS production, inhibited NLRP3 inflammasome activation and reduced inflammatory cytokine levels (Jiang et al., 2016). *In vitro*, the LPS-producing *E. coli* O157:H7 induced significant upregulation of NLRP3 assembly along with caspase-1 activation and oxidative stress. Quercetin protected NLRP3 activation upon *E. coli* infection in Caco-2 epithelial cells demonstrating its potential to protect the GIT epithelial barrier against pathogenic insults (Xue et al., 2017). Another study suggested that quercetin specifically inhibited NLRP3, and not NLRP1, inflammasome activation by interfering with ASC oligomerization in a dose-dependent manner resulting in lower IL-1β release (Domiciano et al., 2017). Finally, in a streptozotocin-induced diabetes nephropathy model, quercetin suppressed NLRP3 inflammasome activation via, in part, its anti-hyperuricemic effects (Wang C. et al., 2012) demonstrating quercetin’s inflammasome-inhibition action *in vivo*. Apigenin, a natural flavone, normalized the expression levels of NLRP3 and IL-1β following microglial activation caused by chronic unpredictable stress in the prefrontal cortex of rats via upregulation of peroxisome proliferator-activated receptor (PPAR)γ receptors (Li et al., 2016). Grape seed-derived procyanidins, rich in apigenin, significantly attenuated gout pain in CD-1 mice caused by macrophage-mediated inflammation and inflammasome activation (Liu H.J. et al., 2017). Finally, in macrophages, apigenin was shown to inhibit LPS-induced production of cytokines primarily through the inhibition of caspase-1 activity and disruption of the NLRP3 inflammasome assembly as well as inhibiting ERK1/2 activation (Zhang et al., 2014).

Several polyphenols have been shown to attenuate the onset of sterile inflammatory cascades. For example, resveratrol can normalize P2X7R expression in a model of chronic pain in rats (Wu et al., 2017). Resveratrol was also shown to exhibit a hepatoprotective effect in diabetic rats mostly through the modulation of RAGE receptor expression (Khazaei et al., 2016). *In vitro*, GSPE attenuated the advanced glycation end products (AGE)-modified bovine serum albumin insult in HUVEC cells by attenuation of surface RAGE expression (Zhang et al., 2013). A GSPE extract was also shown to reduce encephalopathy associated with chronic diabetes through modulation of the AGE/RAGE/NF-κB pathway in the hippocampus (Lu et al., 2010). In a long-term high-fructose fed model of neurodegeneration in rats, therapeutic supplementation with 6% polyphenol rich grape powder for 12 weeks reduced RAGE expression and tau hyperphosphorylation (Liao et al., 2017). Apigenin and diosmetin, both grape-derived polyphenols, potently and dose-dependently inhibited AGE-induced nitric oxide and TNFα release (Chandler et al., 2010). Finally, there has been some evidence suggesting that quercetin can modulate the RAGE/NF-κB cascade as quercetin attenuated atopic dermatitis symptoms, including downregulation of cytoplasmic HMGB1, RAGE and nuclear NF-kB translocation (Karuppagounder et al., 2015).

### Tryptophan Metabolism

Serotonin depletion is one of the most robust blood markers of severe depression (Anderson et al., 1990) and the classical “serotonin hypothesis” describes how diminished serotonin levels play a causative role in depressive phenotypes. However, this hypothesis has been routinely challenged in recent years as the serotonin hypothesis has failed to be substantiated. Diets depleted in tryptophan, the precursor to serotonin synthesis, fail to show any alterations in mood in healthy participants (Smith et al., 1997) indicating that reduced serotonin is neither necessary nor sufficient to cause depression. If this is true, then why are reduced levels of plasma serotonin such a strong biochemical marker of depression? The answer may be the reallocation of tryptophan toward its pro-inflammatory kynurenine degradative pathway, a transition that is dependent on the gut microbiota.

There are two competing pathways for tryptophan metabolism, the methoxyindole and kynurenine pathways. Along the methoxyindole pathway, only 1–5% of dietary tryptophan is synthesized into serotonin, which occurs namely in the enterochromaffin cells in the GIT tract, producing 95% of the body’s serotonin (Gershon and Tack, 2007). In the GIT, serotonin is responsible for controlling motility, secretion and absorption of nutrients, intestinal transit time and colonic tone. Approximately 10–20% of the tryptophan allocated toward serotonin development will directly pass through the BBB initiating serotonin synthesis in the brain (Gal and Sherman, 1980). The remaining tryptophan is metabolized along the kynurenine pathway, which forms several metabolites important for the pathophysiology of depression. The balance of tryptophan metabolism is determined by the activation of the rate-limiting enzymes of kynurenine production, which under normal physiological conditions is controlled by the availability of tryptophan itself and the kynurenine pathway remains stabilized (Cervenka et al., 2017). However, under pathophysiological conditions, elevated inflammation and stress can disrupt the balance of kynurenine production.

The rate-limiting enzymes of tryptophan metabolism are indoleamine- 2,3-dioxygenase (IDO) found in all extrahepatic tissues including the brain and tryptophan-2,3-dioxygenase (TDO) found in the liver. Of particular importance, IDO, inducible by IFNγ, is found in the astrocytes, microglia, endothelial cells and macrophages (Gal and Sherman, 1980). TDO, however, is more heavily influenced by corticosteroids produced by the stress response (O’Mahony et al., 2015) linking HPA activation with tryptophan metabolism. There are two competing pathways that further metabolize kynurenine and the resultant metabolites, namely kynurenic acid (KA) and quinolinic acid (QA), are potent neuro- and immuno-modulatory factors. KA is regarded as neuroprotective as its primary function is to antagonize the glycine co-agonist site on NMDA receptors to prevent excitotoxicity (Kessler et al., 1989). On the other hand, QA is neurotoxic, agonizing the same site on the NMDA receptors promoting excitotoxicity (Guillemin, 2012). In the brain, KA is mostly produced in the astrocytes while QA by the microglia and macrophages (Guillemin et al., 2005). Under a state of chronic inflammation, the elevation in corticosterones and inflammatory cytokines increase the peripheral and central production of kynurenine, consequently reducing serotonin production in the brain (Li et al., 2017). In addition, proinflammatory cytokines activate the enzyme kynurenine-3-monooxygenase, which shifts the metabolism of kynurenine from KA to QA increasing the production kynurenine’s more neurotoxic downstream metabolites.

Elevated brain QA has been recorded in brains of patients with inflammatory neurological diseases (Stone, 2001) and in depressed patients that attempted suicide for up to 2 years after their attempt (Bay-Richter et al., 2015). In the serum of patients with major depression, there is a reduced ratio of KA to QA (Savitz et al., 2015) associated with an inverse correlation to hippocampal volume, a canonical marker of MDD. Additionally, one of the intermediates between kynurenine and QA, 3-hydroxykynurenine, a potent free-radical generator, directly causes neuronal apoptosis, in addition to activating inflammasome activity (Okuda et al., 1998).

Unfortunately, the development of pharmaceutical interventions to modulate the kynurenine pathway have been unsuccessful. Blocking the activity of IDO or TDO enzymes will leave too much circulating tryptophan to potentially toxic levels, while blocking kynurenine-3-monooxygenase to prevent QA production will skew the KA/QA balance too in favor of KA, which can reduce overall NMDA receptor activity. Further, modulation of IFNγ, or other activators of IDO or TDO, is not specific, and will have widespread side effects (reviewed in Jeon and Kim, 2017). Based on the limitations of pharmacological intervention for kynurenine pathway activity, strategies utilizing the gut microbiota and its ability to produce microbial polyphenolic metabolites may prove successful.

### Tryptophan and the Gut Microbiota

The availability of tryptophan is dependent both on diet and importantly, the composition of the gut microbiota as some species utilize tryptophan for the local synthesis of serotonin while others break it down with their endogenous tryptophanase enzyme into the microbial metabolite indole (O’Mahony et al., 2015). Indeed, germ-free animals have elevated circulating tryptophan levels (El Aidy et al., 2012) and elevated circulating tryptophan is associated with increased serotonin levels in the hippocampus (Clarke et al., 2013). Interestingly, the tryptophanase activity of *B. fragilis* was linked to the pathology of autism spectrum disorders (Hsiao et al., 2013). In another study, administration of *B. infantis* resulted in reduced serotonin metabolite (5-HIAA) concentrations in the frontal cortex (Desbonnet et al., 2008). Further, *L. johnsonii* reduced serum kynurenine concentrations by 17% while correspondingly elevating serotonin levels by 1.4-fold, a result associated with the ability of *L. johnsonii* to suppress IDO activity (Valladares et al., 2013).

Serotonin production in the GIT tract directly connects the gut to neurological signaling as approximately 90% of the dietary tryptophan is metabolized along the kynurenine pathway (O’Mahony et al., 2015), which has a dramatic impact on central serotonin availability. As such, studies have shown that peripherally produced serotonin has neuroactivity, which is critical in many neuropsychiatric conditions including depression (O’Mahony et al., 2015). Interestingly, a fecal microbiota transplant from patients with major depression into germ-free rats induced alterations in tryptophan metabolism, anhedonia and anxiety-like behavior (Kelly et al., 2016) directly linking the gut microbiota composition to depressive-like symptoms.

#### Polyphenols Impacting Tryptophan Metabolism

There are several instances where polyphenols or their metabolites were shown to modulate signaling through the kynurenine pathway. A bolus dose of resveratrol (5 g) in humans significantly reduced tryptophan levels 2.5 and 5 h after treatment in healthy volunteers resulting in a 1.33- and 1.30-fold increased the in kynurenine to tryptophan ratio, respectively (Gualdoni et al., 2016). However, in a preclinical study, neither IDO activity nor serotonin levels were correlated with resveratrol-mediated protective effects on social-stress-induced cytokine release or depressive-like behavior (Finnell et al., 2017). Polyphenols present in black tea, notably catechins and epicatechins, increased kynurenine levels in healthy volunteers resulting in a higher kynurenine to tryptophan ratio (Gostner et al., 2015). Similarly, EGCG dose-dependently inhibited IDO mRNA and protein expression in human colorectal cells, in correlation with reduced IFNγ levels, possibly through modulating the phosphorylation status and hence activity of STAT1 (Ogawa et al., 2012). In contrast, a group of flavone polyphenols where shown to inhibit IDO activity, but not mRNA expression, in human neuronal stem cells with apigenin having the greatest inhibitory activity and genistein and quercetin the lowest (Chen et al., 2012). It is clear that polyphenols impact tryptophan metabolism; however some of the effects seem inconsistent, likely due to the variable bioavailability of the polyphenols dependent on the composition of the microbiota. Nevertheless, development of synbiotic strategies to optimize the production of polyphenolic metabolites may successfully modulate the activity of the kynurenine pathway to regulate serotonin levels in the brain of depression patients.

### Neurogenesis and Synaptic Plasticity in Depression

Most animal models of depression are focused on stress-induced inflammatory models that result in neurodegeneration of specific brain areas and consequently, a depressive phenotype. However, under natural conditions, although neuroinflammation does play a major role in depression, reduced neurogenesis is another major pathological concern (Mahar et al., 2014). Many groups believe that suppressed neurogenesis leads to depression (Kim, 2016) and that this fact is underestimated based on the use of animal models as adult neurogenesis in humans is higher compared to rodents (Spalding et al., 2013). Chronic stress impairs hippocampal neurogenesis, which consequently impacts HPA axis regulation (Dranovsky and Hen, 2006). This feedforward mechanism exacerbates affective behavioral responses, while predisposing an individual to subsequent depressive episodes (Mahar et al., 2014). Indeed, elevated microglial activity is associated with reduced hippocampal neurogenesis, which could account for the canonical loss of hippocampal volume associated with depression (Kempermann, 2002).

BDNF is a neurotrophic factor that is a key modulator of hippocampal neurogenesis. BDNF binds to the tropomyosin receptor kinase B (TrkB) whose downstream signaling pathways play an important role in the structural plasticity induced by depression. Several studies have implicated BDNF levels in multiple brain areas with the pathophysiology of depression with decreased levels in the dentate gyrus and the CA3 of the hippocampus and prefrontal cortex or elevated levels in the nucleus accumbens promoting depressive phenotypes (reviewed in Zhang et al., 2016). As such, TrkB receptor agonists such as 7,8-dihydroxyflaone and receptor antagonists such as ANA-12 have antidepressant effects (Jang et al., 2010), which indicates the sensitivity of physiology to variable levels of BDNF. In a chronic unpredictable stress model of depression, depressive symptoms were correlated to reduced BDNF levels in the hippocampus resulting in the mounting decrease in hippocampal CA1 pyramidal neurons (Qiao et al., 2017). Studies have shown that peripheral levels of BDNF can stimulate overall hippocampal neurogenesis (Schmidt and Duman, 2010) indicating that peripheral physiological effects, such as that mitigated by the gut microbiota, could potentially have antidepressant effects through modulating neurogenesis.

Indeed, there is evidence suggesting that the gut microbiota can alter the expression of neurotrophins such as BDNF in the hippocampus and proteins involved in their synaptic transmission such as synaptophysin and PSD-95 in the striatum (Bercik et al., 2011). Treatment of post-weaned mice with antibiotics was shown to reduce anxiety-like behaviors while promoting cognitive deficits and significantly reducing BDNF levels in the adult brain (Desbonnet et al., 2015). Similarly, depleted microbiota in adult mice also lead to significant depletion of BDNF in the brain, associated with greater susceptibility to depressive-like phenotypes (Hoban et al., 2016). All of these studies indicate that the gut microbiota plays a significant role in managing the levels of BDNF in the brain; however only a handful of studies have investigated how supplementation with probiotics can alter these levels. In one study, supplementation with *L. helveticus* NS8 reduced restraint-stress induced behavioral and pathophysiological markers of depression, specifically including elevated levels of hippocampal BDNF (Liang et al., 2015). In an aged model of Fisher rats, *L. pentosus* var. *plantarum* C29 restored age-reduced loss of motor activity and reduced BDNF levels, while simultaneously ameliorating variations of Akt, mTOR and NF-κB in the hippocampus (Jeong et al., 2015). Similarly, in aged mice, *L. brevis* OW38 reduced the associated inflammaging and increased the spontaneous alternation behavioral phenotype through the restoration of BDNF expression (Jeong et al., 2016). Recently, C57BL/6J mice subjected to a 5-week chronic unpredictable stress paradigm were supplemented with *Bifidobacterium longum* subsp. *infantis* E41 and *Bifidobacterium breve* M2CF22M7, which together reduced the depressive phenotype partially through rescuing BDNF levels in the brain (Tian et al., 2019). A similar study also showed that supplementation with *Clostridium butyricum* could reduce the depression phenotype and reduced BDNF levels in male C57BL/6J mice undergoing the same stress paradigm (Sun et al., 2018). In contrast, in a randomized controlled clinical trial, 79 patients with moderate scores of self-report mood measures were allocated to take a mixture of *L. helveticus* and *B. longum* for 8 weeks. Although there was significant improvement in the depression score (60%), there was no variation in several plasma biomarkers including BDNF (Romijn et al., 2017) indicating that there is a complex relationship between the composition of the gut microbiota and its effect on neurogenesis and neuroplasticity metabolites.

Likewise, a positive relationship between the consumption of polyphenols with markers of neurogenesis including BDNF has been observed. Several polyphenol-rich natural extracts have been shown to be key modulators of neuroplasticity (Sangiovanni et al., 2017) while many isolated polyphenols have been shown to promote neurite outgrowth *in vitro* including resveratrol, EGCG, ferulic acid, caffeic acid and quercetin derivatives, through mechanisms involving BDNF activity (reviewed in Moosavi et al., 2016). A low-dose unfractionated green tea polyphenol preparation (<0.1 μg/ml) or a low-dose of one of its active ingredients EGCG (<0.5 μM) potentiated the neuritogenic ability of a low concentration of BDNF in PC12 cells (Gundimeda et al., 2014). In an oxidative stress model of anxiety in rats, GSPE (15 g/L/day) treatment over 3 weeks significantly reduced anxiety-like behavior while restoring, among other markers, BDNF levels indicating that oxidative-stress induced changes in behavior can be rescued by grape seed polyphenol treatment (Allam et al., 2013). In a rat model of posttraumatic stress with a single-prolonged stress through foot shock, grape powder administered at 15 g/L for 3 weeks following the stress protocol reduced anxiety-like behavior while preventing the loss of BDNF levels in the amygdala of affected animals (Solanki et al., 2015). Finally, in a human intervention study, subjects were given a single dose of whole coffee fruit concentrate powder, green coffee caffeine powder, grape seed extract powder or green coffee bean extract powder. It was found that the grape seed extract powder and the green coffee caffeine powder increased the levels of BDNF in the serum by 31% while the whole coffee fruit concentrate powder increased BDNF levels by 143% (Reyes-Izquierdo et al., 2013). As indicated earlier in this review, all of these extracts are modulated by the gut microbiota. It can therefore be predicted that there is a synergistic impact of the dietary polyphenols with the gut microbiota in modulating the plasma and presumably peripheral and central levels of BDNF.

## Conclusion

**FIGURE 3:**
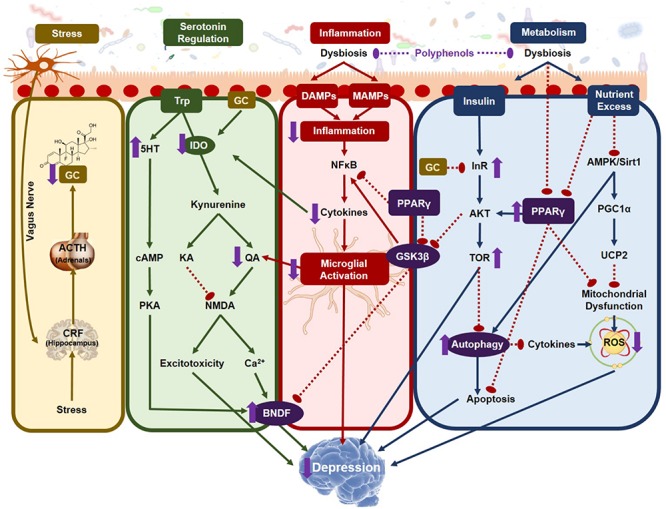
Mechanisms connecting gastrointestinal dysbiosis with biological signature of depression. Depression is a multifaceted disorder with several coordinating pathologies, most which can be modulated by gut microbiota modifying agents including dietary polyphenols. The main gut-brain-axis mechanisms through which polyphenols and their microbial-derived metabolites can elicit a positive effect on depression are stress (yellow), serotonin regulation (green), inflammation (red) and metabolism (blue), effects which are shown as purple arrows. GC, glucocorticoids; ACTH, adrenocorticotropic hormone; CRH, corticotropin releasing hormone; 5HT, serotonin; Trp, tryptophan; IDO, indolamine-2,3-dioxygenase; KA, kynuric acid; QA, quinolinic acid; cAMP, cyclic adenosine monophosphate; PKA, protein kinase A; BDNF, brain derived neurotrophic factor; DAMPs, danger associated molecular patterns; MAMPs, microbe associated molecular patterns; PPARγ, peroxisome proliferator activated receptor gamma; GSKβ, glycogen synthase kinase 3 beta; InR, insulin receptor; AKT, Protein kinase B; AMPK, 5′ AMP-activated protein kinase; PGC1, proliferator-activated receptor gamma coactivator; UCP2, uncoupling protein 2; ROS, reactive oxygen species.

Depression is a multifactorial disorder reflecting an accumulation of several pathophysiological conditions including neuroinflammation, elevated microglia activation, an imbalance of tryptophan metabolites and altered BDNF levels. Due to its complexity, no single pharmacological agent targeting one specific aspect of depression’s etiology would be sufficient to ameliorate such a diverse set of risk factors. Recently the gut microbiota’s interaction with dietary polyphenols has been shown to produce a large battery of bioactive metabolites with the ability to simultaneously modulate the multiple risk factors of depression. As each of the microbial-derived bioactive metabolites produced by a single polyphenol-rich botanical have the potential to overlap or complement the bioactivity of other metabolites produced by the same botanical, the possibility of synergistic and multiplexed activity against multiple depression risk factors is enhanced (Figure 3). As demonstrated in this review with the support of mechanistic studies, this synbiotic approach may instigate a paradigm shift in the treatment regime of depression as probiotic and polyphenol-rich botanical supplementation is a cost-effective, long-term treatment option with limited side effects that may be more robust that traditional pharmacological paradigms that target specific depression risk factors.

## Author Contributions

SW conceived, outlined, and prepared the manuscript. GP directed in all aspects of the manuscript’s preparation.

## Conflict of Interest

The authors declare that the research was conducted in the absence of any commercial or financial relationships that could be construed as a potential conflict of interest.
